# Comparative Analysis of Ki-67 Proliferation Index in Colorectal Adenomas and Adenocarcinomas: An Immunohistochemical Study

**DOI:** 10.7759/cureus.106099

**Published:** 2026-03-30

**Authors:** Yengkhom Daniel Singh, Vinutha Gali, Mary Lilly S

**Affiliations:** 1 Pathology, Yenepoya Medical College Hospital, Mangalore, IND; 2 Pathology, Sree Balaji Medical College and Hospital, Chennai, IND

**Keywords:** adenocarcinoma, adenoma, colorectal, immunohistochemistry, ki-67

## Abstract

Introduction: Colorectal carcinoma (CRC) is one of the most common cancers in various parts of the world. The evolution of CRC follows a stepwise genetic acquisition of mutations and a resultant transition from normal mucosa to adenoma and, ultimately, adenocarcinoma. The goal of the study was to assess the different immunohistomorphologic characters in adenomas and adenocarcinomas using the Ki-67 tumor proliferation marker that may have diagnostic or prognostic relevance.

Materials and methods: This descriptive study was conducted on 40 paraffin-embedded tissue blocks collected from the colorectal tissues in a tertiary care hospital in Chennai, Southern India. Ki-67 proliferation index (PI) was analyzed by immunohistochemically stained slides by a set of two pathologists to eradicate bias.

Results: Tissue samples from 25 (62.5%) male and 15 (37.5%) female patients with a mean age of 56.9 ± 16.09 (age range six to 83 years) and a median age of 60 years were included in the study. Adenocarcinomas comprised 24 (60%), whereas 16 (40%) cases were adenomas. There was a significant mean difference between adenomas (Mean PI = 30.97 ± 11.44) and adenocarcinomas (Mean PI = 46.33 ± 25.16) when compared with Ki-67 PI (%), with a p-value = 0.02 (< 0.05).

Conclusions: The Ki-67 immunohistochemical technique is a simple and reproducible technique. A gradual increase in expression of Ki-67 was observed from low-grade adenomas to high-grade adenomas and adenocarcinomas, which may serve as a routine tumor proliferation marker that can be routinely used as a diagnostic or prognostic marker in CRCs.

## Introduction

Colorectal carcinoma (CRC) is the third most common cancer in terms of incidence (1,926,425 cases, 9.6% of total) and the second most common cause of deaths due to cancer (904,019 deaths, 9.3% of total) worldwide. The highest incidence rates of CRC belong to European countries, Australia, New Zealand, North America, and a few Asian countries. Gender distribution shows a higher incidence of colorectal cancers (10.4% in males; 8.9% in females) in males. However, mortality rates follow an opposite trend (9.2% in males; 9.4% in females) among all cancer-related deaths. In India, CRC stands as the fourth most common cancer among males and the fifth most common among females in terms of incidence and the seventh most common cause of cancer-related deaths [1]. 

Multiple modifiable and non-modifiable risk factors, family history, comorbidities, and irradiation contributing to the development of CRCs have been identified in the past. Screening helps detect illness in asymptomatic people early on, with the goal of bettering outcomes. Early-stage CRC has a much better prognosis than more advanced disease, so it lends itself well to screening. Furthermore, the transition from normal mucosa to adenomas or adenomatous polyps and to adenocarcinoma is thought to take a decade or more [2]. As a result, detection and removal of such preneoplastic lesions will help minimize the risk of transformation to CRC. Patients with CRCs may present with a variety of symptoms, such as rectal bleeding, altered bowel habitus such as diarrhea or constipation, anemia due to chronic blood loss, and vague abdominal pain [3].

As the most significant factor in cancer patients, aggressive metastatic activity is primarily caused by changes in the molecular characteristics of tumor cells, including cell growth disruption and regulation of proliferation [4]. Different oncogenes, tumor suppressor gene-related biomarkers, growth factors, angiogenesis, and cell proliferation factors are used in cancer diagnosis and prognosis.

Currently, various screening guidelines in the US are all based on stool-based tests such as fecal immunochemical test (FIT), fecal occult blood test (FOBT), and multi-target stool DNA test (mtDNA), along with adjunct invasive procedures such as colonoscopy, flexible sigmoidoscopy (FS), and computed tomography colonography (CTC) [5-7]. In addition to the limited use of such techniques in developing and underdeveloped countries, certain challenges are also being faced due to tailored screening strategies, decoherent relative risk with absolute risk, a substantial overlap in the range of risk scores between those with advanced neoplasia vs. those without, and other reasons [8].

In 1989, Porschen et al. first measured the cell proliferation on cryostat sections of 61 resected colorectal adenocarcinomas and concluded that Ki-67 nuclear staining is a reliable and reproducible method of determining tumor proliferation in CRCs [9].

In the last two decades, many studies have highlighted the probability of Ki-67 immunostaining as a screening methodology. A descriptive study was conducted on 40 formalin-fixed paraffin-embedded (FFPE) tissue blocks using the nuclear proliferation marker Ki-67 in colorectal tissues at a tertiary care hospital. The goal of the study was to assess the different immunohistomorphologic characters in adenomas and adenocarcinomas using Ki-67 immunohistochemistry (IHC) and to test its eligibility as a routine tumor proliferation marker that may have diagnostic or prognostic relevance. 

## Materials and methods

Study design and setting

This descriptive study was conducted on 40 FFPE tissue blocks of neoplastic colorectal tissues in a tertiary care hospital in Chennai, Southern India. All these blocks were selected through the convenience sampling method based on strict inclusion (colonoscopic biopsies and surgically resected specimens, diagnosed as adenoma and adenocarcinoma on histopathology) and exclusion (cases that initiated prior treatment, chemo/radiotherapy, or were previously operated on) criteria from the archives of the Pathology Department, Sree Balaji Medical College and Hospital, Chennai, for a period of two years. The selected colorectal blocks according to the histopathological diagnosis were classified into two groups: adenomas (n = 24) and adenocarcinoma (n = 16). Before conducting IHC, all samples were re-evaluated by an expert pathologist for histopathological confirmation. Tissues with autolysis, insufficient neoplastic tissue, inflammatory lesions, non-epithelial neoplasms, and post-surgery, chemotherapy, or radiotherapy samples were excluded from the study. Institutional Ethical Committee (IEC) approval from Sree Balaji Medical College and Hospital was taken before the conduct of this study, bearing the IEC no. 002/SBMC/IHEC/2018/1160. 

Immunohistochemical staining procedure

IHC staining was performed according to the manufacturer’s instructions. Thin tissue sections (3 μm) were cut using a rotary microtome (Leica, Germany) from the FFPE blocks from the colorectal area. The sections were placed on poly-L-lysine (PathnSitu Biotechnologies Pvt. Ltd., USA)-coated slides. The tissue sections were then deparaffinized in xylene (two times × two minutes) and rehydrated with descending degrees of ethanol (two times × two minutes). Antigen retrieval was performed by placing the container of the slides in TRIS buffer solution (pH = 9) for 20 minutes at 120°C in a pressure cooker. The slides were cooled at room temperature and deionized in phosphate-buffered saline (PBS). A blocking reagent was added to the sections for ten minutes. Only cleaning and no washing of slides were done. Next, the primary antibody was added to the respective slides, and they were incubated at room temperature inside a humidity chamber for 30 minutes. They were washed with PBS as before. A mouse monoclonal Ki-67 antibody (Clone GM010; PathnSitu Biotechnologies Pvt. Ltd.) was then added (using a dilution of 1:100) to the slides and incubated in a humidity chamber for 30 minutes. They were washed again with PBS as before. DAB chromogen was added and kept for ten minutes, and then it was washed using distilled water. Counterstaining with hematoxylin was done for one minute. All slides were washed in running tap water for five minutes. Dehydration using alcohol was done as 50% alcohol × one minute, 70% alcohol × one minute, and 90% alcohol × one minute. The positive control used for comparison was normal human tonsil tissue.

Ki-67 IHC scoring method

For the purpose of scoring the Ki-67 proliferation index (PI) or labeling index (LI), the immunohistochemically stained slides were analyzed by a set of two trained pathologists to eradicate bias. Inter-observer agreement between the two pathologists was evaluated by Kappa (κ) Agreement (Landis and Koch guideline) and scored as < 0 poor, 0-0.20 slight, 0.21-0.40 fair, 0.41-0.60 moderate, 0.61-0.80 substantial, and 0.81-1.00 almost perfect. The area for our analysis was chosen at the edge of the tumor, where a higher number of well-stained tumor cell nuclei were observed. This may be attributed to the fact that when a tumor grows in size, it grows from the edges of the lesion rather than the expansion of the center of the lesion. These are the so-called “hot spots” with >50% staining. A ≥3 foci, under high power (400x), are visualized to count the number of fully stained and intact nuclei of only the tumor area. Non-neoplastic areas, necrotic areas, thick areas, and overlapping and partially stained nuclei of the tumor cells were avoided during the evaluation. The intensity of staining is not relevant in Ki-67 immunostaining. A minimum of 500 nuclei to a maximum of 1000 nuclei were counted using 400x magnification in such areas for calculating the percentage (%) of stained nuclei [10]. There is no established cut-off score implicated in CRC to stratify the PI. Ki-67-labeled slides from urothelial carcinoma were used as positive control slides.

Statistical analysis

For the purpose of statistical analysis and comparison, data were represented by ‘mean rank’ and then correlated with the aforementioned variables. IBM SPSS Statistics for Windows, Version 23 (Released 2015; IBM Corp., Armonk, New York, United States) was used for analysis, and data were recorded in Microsoft Excel (Microsoft Corporation, Redmond, Washington, United States).

To detect an association between two categorical variables, chi-square tests are applied. To find the mean difference between the groups when compared with the outcome variable, a non-parametric Kruskal-Wallis test is used as an analog for a novel parametric test. The Mann-Whitney test is used to compare two independent groups that differ in distribution. Both are nonparametric tests used to determine whether there is a statistically significant difference between groups. A p-value < 0.05 is considered significant throughout the study.

## Results

**Figure 1:**
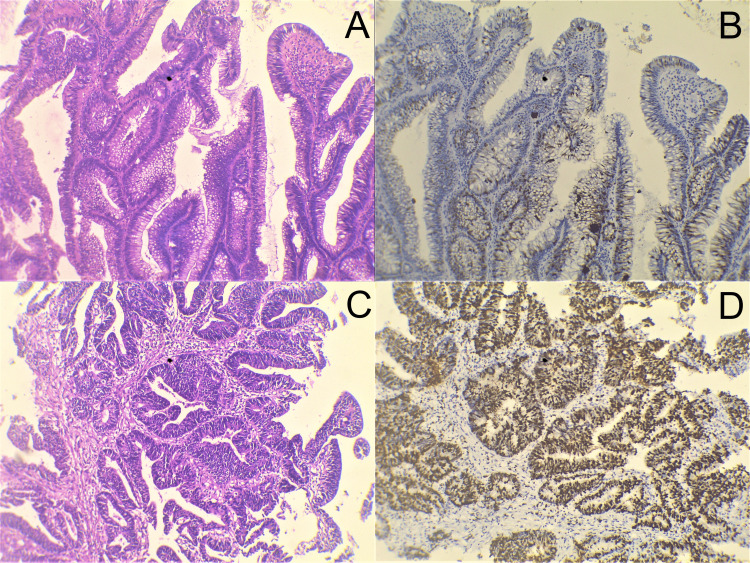
Ki-67 immunohistochemical findings. A: H&E (100x), a villous adenoma showing predominant villous morphology; B: Ki-67 (MIB1) IHC (100x), villous adenoma showing low proliferative activity; C: H&E (100x), well-differentiated (grade I) adenocarcinoma with well-formed malignant glands; D: Ki-67 (MIB1) IHC (100x), well-differentiated (grade I) adenocarcinoma showing high proliferative activity.

**Figure 2:**
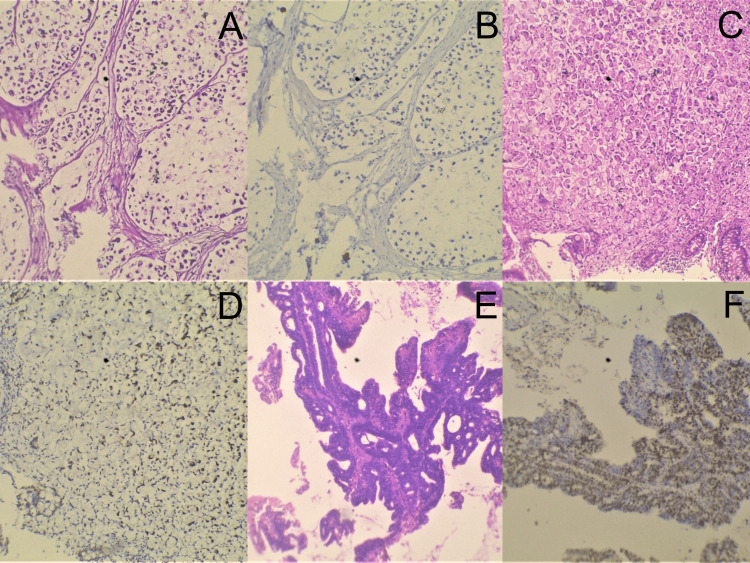
Heterogeneous Ki-67 immunohistochemical findings. A: H&E (100x), mucinous carcinoma showing mucin-secreting tumor cells in pools of extracellular mucin; B: Ki-67 (MIB1) IHC (100x), mucinous carcinoma showing very low proliferative activity; C: H&E (100x), signet ring cell adenocarcinoma showing diffuse sheets of signet ring-shaped tumor cells with abundant intracytoplasmic mucin; D: Ki-67 (MIB1) IHC (100x), signet ring cell adenocarcinoma showing low proliferative activity; E: H&E (100x), adenoma-like adenocarcinoma showing villous-like architecture of the malignant glands; F: Ki-67 (MIB1) IHC (100x), an adenoma-like adenocarcinoma showing moderate proliferative activity.

A total of 40 cases were enrolled in the present study. Tissue samples from 25 (62.5%) male and 15 (37.5%) female patients with a mean age of 56.9 ± 16.09 (age range six to 83 years) and a median age of 60 years were included in the study. Table 1 shows the age and gender distribution of all cases. Twenty-four (60%) out of the 40 cases turned out to be adenocarcinomas on histopathologic examination, whereas 16 (40%) cases were adenomas. Mean ages for females with adenomas and adenocarcinoma lesions were 61.35 ± 13.14 years and 57.31 ± 16.09 years, respectively, whereas median ages were 64 years and 60 years, respectively. Mean ages for males with adenomas and adenocarcinomas were 58 ± 16.41 years and 56.56 ± 16.16 years, respectively, whereas median ages were 60 years each. The majority of CRC cases were adenocarcinoma, not otherwise specified (NOS) type, with 17 (42.5%) cases, among which 14 (66.7%) cases belonged to moderately differentiated adenocarcinoma, being the most common histologic grade. There was moderate κ-agreement between the Ki-67 PI scoring in adenomas (mean = 30.97 ± 11.44) and substantial agreement in Ki-67 PI (mean = 46.33 ± 25.16) scoring in adenocarcinomas. Table 2 shows the various lesions based on the sites of their occurrences. Ki-67 IHC displayed marked differences in staining and, hence, PI (%), between adenomas and adenocarcinoma (Figure 1). There was a significant mean difference between adenomas and adenocarcinomas when compared with Ki-67 PI (%), with a p-value = 0.02 (<0.05) (Table 3). A significant difference was seen between Ki-67 PI (%) and histological grades of adenoma lesions with a p-value = 0.003 (<0.05) (Table 4). Similarly, a significant correlation was seen in adenocarcinomas between Ki-67 PI (%) and histological grades with a p-value = 0.03 (<0.05) (Table 5). There were five (12.5%) cases belonging to mucinous carcinoma, and a case each belonging to signet ring cell adenocarcinoma (2.5%) and adenoma-like adenocarcinoma (2.5%), respectively. A heterogeneous Ki-67 IHC was seen in these special histopathological subtypes of adenocarcinoma (Figure 2). 

**Table 1:** Age and gender distribution of the cases.

Age interval			
Age in years	Frequency		Percentage (%)
	Males	Females	
≤ 40	7	0	17.5
41-50	2	3	12.5
51-60	9	1	25
61-70	4	9	32.5
71-80	1	2	7.5
>80	2	0	5.0
Total	25 (62.5%)	15 (37.5%)	100.0

**Table 2:** Distribution of the various histopathological types of lesions based on sites of occurrence. NOS: not otherwise specified

Sites	Juvenile polyp	Tubular adenoma	Villous adenoma	Tubulovillous adenoma	Adenocarcinoma NOS	Mucinous adenocarcinoma	Signet ring cell adenocarcinoma	Adenoma-like adenocarcinoma	Total
Cecum	0	0	1 (2.5%)	1 (2.5%)	0	1 (2.5%)	0	0	3 (7.5%)
Ascending colon	0	2 (5%)	0	0	7 (17.5%)	0	0	1 (2.5%)	10 (25%)
Hepatic flexure	0	0	0	0	0	1 (2.5%)	0	0	1 (2.5%)
Transverse colon	0	1 (2.5%)	0	0	2 (5%)	0	1 (2.5%)	0	4 (10%)
Splenic flexure	0	0	0	0	0	1 (2.5%)	0	0	1 (2.5%)
Descending colon	0	0	0	1 (2.5%)	0	0	0	0	1 (2.5%)
Sigmoid colon	0	2 (5%)	0	2 (5%)	3 (7.5%)	2 (5%)	0	0	9 (22.5%)
Rectum	1 (2.5%)	0	4 (10%)	1 (2.5%)	5 (12.5%)	0	0	0	11 (27.5%)
Total	1 (2.5%)	5 (12.5%)	5 (12.5%)	5 (12.5%)	17 (42.5)	5 (12.5%)	1 (2.5%)	1 (2.5%)	40 (100%)

**Table 3:** Mann-Whitney test for correlation of Ki-67 and lesion type. There is a significant mean difference between benign and malignant when compared with Ki-67 LI (%) with a p-value <0.05 (0.02).

Ranks				
Lesion type		N (mean Ki-67 ± SD)	Mean rank	Sum of ranks
Ki-67 LI (%)	Benign	16 (30.97 ± 11.44)	16.84	269.50
	Malignant	24 (46.33 ± 25.16)	22.94	550.50
	Total	40		

**Table 4:** Kruskal-Wallis test for correlation of Ki-67 and histologic grades of adenomas. There is a significant difference between diagnosis when compared with Ki-67 LI (%) according to histological grades, with a p-value <0.05 (0.003). * Includes a case of juvenile polyp with low-grade dysplasia.

Histological grade		Diagnosis	N (mean Ki-67 ± SD)	Mean rank
Low-grade dysplasia	Ki-67 LI (%)	Tubular adenoma*	6 (19.04 ± 10.44)	3.40
		Tubulo-villous adenoma	1 (28.5 ± 0)	4.00
		Total	7	
High-grade dysplasia	Ki-67 LI (%)	Tubulo-villous adenoma	4 (38.23 ± 1.01)	5.00
		Villous adenoma	5 (37.28 ± 7.64)	5.00
		Total	9	

**Table 5:** Kruskal-Wallis test showing correlation of Ki-67 and different histologic grades among malignant lesions. There is a significant mean difference between diagnosis when compared to Ki-67 LI (%), with a p-value <0.05 (0.03) between different histological grades among malignant lesions. NOS: not otherwise specified

Histological grade		Diagnosis	N (mean KI-67 ± SD)	Mean rank
NA	Ki-67 LI (%)	Mucinous adenocarcinoma	5 (27.50 ± 22.40)	3.80
		Signet ring cell adenocarcinoma	1 (5 ± 0)	2.00
		Adenoma-like adenocarcinoma	1 (70 ± 0)	7.00
Low-grade (Grade 1, 2)	Ki-67 LI (%)	Adenocarcinoma, NOS	15 (49.83 ± 21.41)	8.00
High-grade (Grade 3)	Ki-67 LI (%)	Adenocarcinoma, NOS	2 (77.50 ± 3.54)	15.75

## Discussion

The proliferation of cells is typically measured by the determination of nuclear protein antigens, the cellular concentration of which increases during the phases preceding the mitotic cycle, where they are rapidly catabolized. The Ki-67 nuclear antigen is a non-histone protein that is present in all phases of the cell cycle, with the exception of phase G0. Ki-67 plays an important role in the proliferation of cells. Ki-67 is used extensively for evaluating proliferating cancer cells due to its high sensitivity [11,12]. Ki-67 has been used as an indicator for the prognosis of well-known malignancies such as gastric cancer, gastrointestinal tract cancer, prostate cancer, and breast cancer in most studies using IHC [13,14]. It is evidenced that MIB-1 (Ki-67) is expressed by all the crypts in adenomatous polyps, more particularly in upper and surface epithelium, whereas it is expressed only by basal and lower-half crypts in non-adenomatous polyps [15]. Various studies showed an escalating increase in Ki-67 protein expression, indicating increased proliferation from normal colon mucosa to adenomatous mucosa and, ultimately, to CRC [16-18]. Additionally, Ki-67 overexpression has also been associated with poor prognosis, and an inverse relationship is seen between Ki-67 and the survival rate of patients with CRCs by Lumachi F et al. [19], Fernández-Cebrián JM et al. [20], Wu XS et al. [21], Furudoi A et al. [22], and Hayashi H et al. [23].

We observed a significant correlation between Ki-67 LI when adenomas were compared with adenocarcinomas (p-value = 0.04). Such a finding was also detected by Lin MX et al. [16], Risio M et al. [24], and Saleh HA et al. [25]. Intercomparison among adenomas showed a significant correlation between lesions with high-grade dysplasia and lesions with low-grade dysplasia (p-value = 0.001). Such a progressive increase in mean Ki-67 LI, from low-grade to high-grade dysplasia, was also seen by Risio M et al. [24].

Similarly, there was a significant correlation between adenocarcinoma with high-grade (grade III) and low-grade (grades I and II) histology (p-value = 0.0234). Heidari Z et al. [12], Ahmed NY et al. [26], Salminen et al. [27], Bhagyalakshmi et al. [28], and Sen A et al. [29] also had similar observations. We also observed that the Ki-67 LI was lower in mucinous carcinoma, signet ring cell adenocarcinoma, and adenoma-like adenocarcinoma compared to adenocarcinoma NOS. Similar observations were also seen by Ahmed NY et al. [26] and Nabi U et al. [30] in their study, with a significant difference between non-mucinous carcinoma and mucinous and signet ring carcinoma.

In concordance with Ahmed NY et al. [26], Salminen et al. [27], and Nabi U et al. [30], we did not find any statistically significant correlations between Ki-67 LI overexpression with the age and gender of patients.

Limitations and future perspectives

The authors should clearly acknowledge certain limitations of the study, such as small sample size, single-center design, convenience sampling, absence of longitudinal outcome or survival data, lack of staging correlation, and limited statistical power for subgroup analyses. Population-specific studies will address the heterogeneity in divergent populations owing to the demography, population growth, aging, unfavorable trends, and differing preventive techniques. Therefore, stratified and larger-scale studies may be required.

## Conclusions

In conclusion, we acknowledged the Ki-67 immunohistochemical technique as a simple and reproducible technique, which is readily applicable in FFPE tissue sections. We observed a higher expression of Ki-67 in adenocarcinomas compared to adenomas. The Ki-67 expression also increased with the increasing histologic grade of adenomas and adenocarcinomas. Hence, the possible utility of Ki-67 immunohistochemistry in screening, diagnosis, and patient monitoring for management of CRCs can slowly but surely be of paramount clinical interest in the future.
